# WS_2_/WO_3_ modified carbon anode as efficient electrocatalysts for enhancing electricity generation and pollution removal

**DOI:** 10.3389/fmicb.2025.1589441

**Published:** 2025-04-28

**Authors:** Yugang Sang, Quantong Jiang, Fang Guan, Nan Wang, Ini-Ibehe Nabuk Etim, Keliang Fan, Jizhou Duan

**Affiliations:** ^1^Department of Materials Science and Engineering, Qilu University of Technology, Jinan, China; ^2^State Key Laboratory of Advanced Marine Materials, Institute of Oceanology, Chinese Academy of Sciences, Qingdao, China; ^3^Guangxi Key Laboratory of Marine Environmental Science, Institute of Marine Corrosion Protection, Guangxi Academy of Sciences, Nanning, China; ^4^Department of Bioengineering, Qilu University of Technology, Jinan, China

**Keywords:** MFCs, nanomaterials, anode modification, electricity generation, pollutant removal

## Abstract

Microbial fuel cells (MFCs) have emerged as a new energy technology to solve severe energy and environmental issues. As a bridge connecting the internal and external circuits and a habitat for microorganisms, the anode is a key component influencing the performance output of MFCs. Recently, tungsten trioxide (WO_3_) and tungsten disulfide (WS_2_) can be used for the MFC setup. In this study, a direct hydrothermal synthesis method was employed to prepare WS_2_/WO_3_ nanomaterials. It was subsequently integrated with carbon paper (CP) to develop WS_2_/WO_3_-CP and WO_3_-CP anodes for MFCs. Contact angle tests showed that the hydrophilicity of the WS_2_/WO_3_-CP electrode was significantly improved. In electrochemical tests, the MFCs with WS_2_/WO_3_-CP anode exhibited lower charge transfer resistance and higher electron transfer efficiency than the original ones. The MFC with the WS_2_/WO_3_-CP anode had a maximum power density reaching 2.32 W·m^−2^, which was 1.34 and 3.09 times higher than that of the WO_3_-CP and bare CP anodes, respectively. Meanwhile, this MFC with the WS_2_/WO_3_-CP anode showed higher removal rates of chemical oxygen demand and SO_4_^2−^ than the WO_3_-CP and CP anodes. The modified WS_2_/WO_3_ nanomaterials are promising materials that can be adopted for MFCs industrial use.

## 1 Introduction

The low availability of energy technology has been one of the major challenges hindering sustainable development. Under the dual challenges of the continuous growth of energy demand and the increasingly severe environmental problems, it has become an urgent task to find sustainable clean energy sources and efficient pollution control technologies (Igliński et al., 2023). Currently, microbial fuel cell (MFC) technology has emerged as an appropriate alternative for energy-positive wastewater treatment and bioelectricity production via bioelectrochemical remediation mediated by electroactive microbes (Verma et al., 2021; Boas et al., 2022).

The MFCs technology relies on electroactive microorganisms (EAMs) to directly convert the chemical energy from organics into electrical energy. Since MFCs have the capability to significantly reduce the substantial energy consumption that stems from the aeration process in wastewater treatment, they are regarded as a highly economically advantageous alternative compared to conventional sewage treatment technologies (Chen et al., 2019; Munoz-Cupa et al., 2021). This economic viability highlights the potential of MFCs in cost-saving and also positions them as a promising solution in the field of environmental protection and sustainable wastewater management. A series of studies on environmental monitoring, biological hydrogen production, metal recovery, and other aspects using MFC have been carried out to improve its practical feasibility (Logan et al., 2006; Selembo et al., 2009; Do et al., 2022; Mkilima et al., 2024). However, due to the nature of extracellular electron transfer (EET) dominated by EAMs in MFCs, the electricity generation of MFCs are easily restricted by microbial metabolic characteristics and surrounding environmental factors such as temperature, pH, substrate concentration, and surface properties of the electrode (Walter et al., 2020; Bird et al., 2022; Sonawane et al., 2022; Ouyang et al., 2023). These restrictive conditions are unfavorable to the improvement of electron transfer, limiting the removal rate of chemical oxygen demand (COD) and the output power density of MFCs being far lower than the actual usage requirements.

Presently, some viable strategies have been proposed for enhancing electricity generation and wastewater treatment, such as electrode modification, precultivation of dominant EAMs, and the optimization of the device structure (Nam et al., 2017; Zheng et al., 2022; He et al., 2024). Among these methods, electrode modification is important in gaining high efficiency. This is due to their excellent electrical conductivity, a diverse array of carbon materials, such as carbon cloth, carbon paper, and carbon felt, have been widely used as substrate materials for MFC anodes. Compared with metal electrodes, these carbon materials generally possess favorable characteristics such as easily functionalized surfaces, excellent biocompatibility, low cost, and high electrical conductivity (Mier et al., 2021). However, these untreated carbon electrodes commonly suffer from problems such as low specific capacitance and poor hydrophilicity (Wang et al., 2009; Zhong et al., 2018). These drawbacks, in turn, give rise to challenges in microbial attachment and EET.

In recent years, transition metal oxides have been regarded as effective options for modifying carbon electrodes due to their wide accessibility, low cost, properties of good catalytic activity, and excellent biocompatibility (Santos et al., 2023). Tungsten oxide (WO_3_) is an inexpensive metal oxide material that has been proven to have good catalytic activity and biocompatibility. Studies showed that the maximum power density of the MFC using WO_3_ as the anode catalytic material was improved by five times compared to that of an MFC with an unmodified electrode (Das and Ghangrekar, 2020). Miran et al. (2021) used a carbon felt electrode modified with Fe_3_O_4_ as the anode material in MFC, and its maximum power density reached 309 mW g·L^−1^, 1.86 times that of a bare carbon felt electrode.

Although those modifications by single transition metal oxides such as MnO_2_, TiO_2_, and NiO improved the electrode performance to a certain extent, their poor intrinsic conductivity limits the electron transfer efficiency and their practical application as a high-performance electrode materials for energy generation (Zhong et al., 2018; Arkin et al., 2023; Santos et al., 2023). To overcome this problem, the combination of different transition metal compounds is considered an effective strategy. Tungsten disulfide (WS_2_), a typical two dimensional transition metal sulfide, has a unique layered structure, excellent electrical properties, and good chemical stability (Mohan et al., 2023). Its layered structure endows it with a large specific surface area, which is beneficial for microbial attachment and electron transfer. Nguyen et al. (2024) prepared WS_2_/WO_3_ nanocomposites by hydrothermal synthesis and used them as catalysts for hydrogen evolution reaction. Wang found that the interfacial electric field of WS_2_/WO_3_ shifted the center of the d-band of W upwards and enhanced the adsorption energy of the intermediate (e.g., the adsorption energy of NH_2_ increased from 1.64 to 2.22 eV), thereby accelerating the reaction kinetics (Wang et al., 2023). In the electrocatalytic nitrogen reduction reaction test, the NH_3_ yield using WS_2_/WO_3_ was 3 times higher than that of pure WS_2_ and 5 times higher than that of WO_3_. two-dimensional structure of WS_2_ and the nanorod structure of WO_3_, the number of active sites and the electrical conductivity of the composites prepared by Shreya et al. were enhanced (Shreya et al., 2024). Their impedance spectroscopy results show that the charge transfer resistance of WS_2_/WO_3_ is 397.7 Ω, which is much lower than that of WO_3_ (1816 Ω).

Thus, it can be shown that the heterostructured WS_2_/WO_3_ has been studied in the fields of electrolysis hydrogen production, photocatalysis, and ammonia synthesis. Whoever, there are very few reports on the application of WS_2_/WO_3_ nanomaterials in MFCs. Therefore, the synergistic effects of combining WS_2_ and WO_3_ are expected to enhance the electrochemical performance of the electrode. In this work, the WS_2_/WO_3_ heterostructure was prepared employing a simple direct hydrothermal synthesis method and is modified onto the carbon paper electrode, which is further used as an anode electrode in MFC. This research aims to explore the feasibility of leveraging the interfacial synergistic effect between WS_2_ and WO_3_ to reduce the charge-transfer resistance, enhance the biocompatibility of the anode, and improve its ability to treat pollutants (including organic pollutants and sulfates) in wastewater. Thus, providing new ideas for the high-efficiency and low-cost MFC anode materials.

## 2 Materials and methods

### 2.1 Chemicals and substrate electrodes

Carbon paper (CP) with a size measuring 1 × 3 cm (TGP-H-060) material was purchased from Toray Industries, Inc. (Japan) and used as the anode substrate. Conductive carbon brushes (CB), with a diameter of 3 cm, brush length of 3 cm, and rod length of 12 cm, were bought from Zhengzhou Yaole Instrument Technology Co., Ltd. (China). The proton exchange membrane (Nafion 117, PEM) between the two electrodes and the Nafion D520 solution (5%) were obtained from DuPont (USA). All the chemicals used for electrode preparation and microbial culture were of analytical reagent (AR) grade and purchased from Sinopharm Chemical Reagent Co., Ltd.

### 2.2 Preparation of WO_3_ and WS_2_/WO_3_ nanoparticle modified electrodes

**Figure 1:**
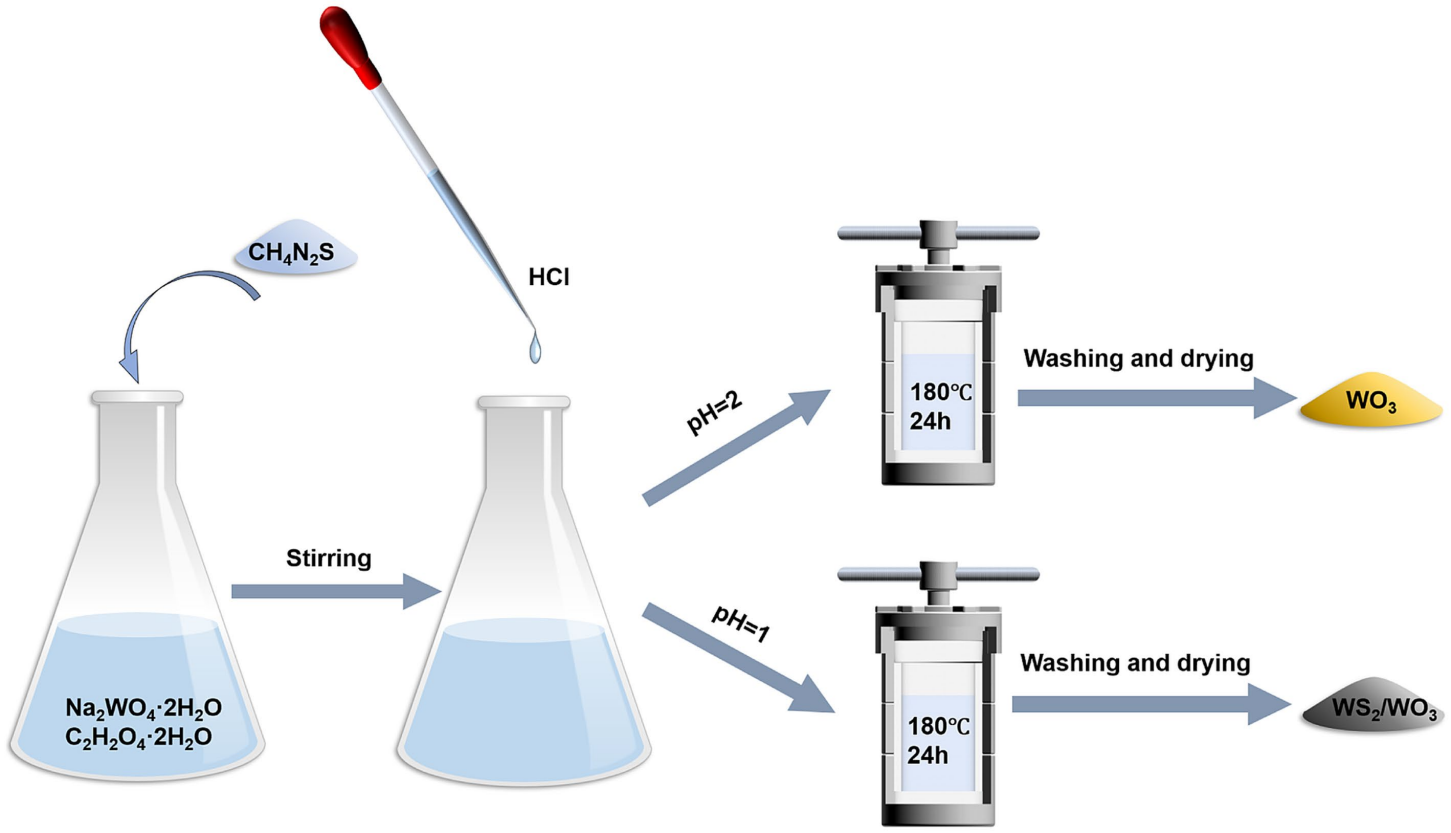
Schematic diagrams of the experimental procedures.

WS_2_/WO_3_ nanoparticles were hydrothermally synthesized by modifying the method described in the previous literature procedure (Liang et al., 2023). First, 1 g sodium tungstate dihydrate (Na_2_WO_4_·2H_2_O, >99%) and 1.26 g Oxalic acid dihydrate (C_2_H_2_O_4_·2H_2_O, >99%) were dissolved in 100 mL deionized water and stirred until completely dissolved. Then, 1.6 g thiourea (CH_4_N_2_S,> 99%) was added to the solution and stirred evenly. Subsequently, the pH of the solution was adjusted to 1.0 with HCl. The prepared solution was transferred into a hydrothermal reaction kettle and kept in a blast drying oven maintained at 180°C for 24 h. The black solid obtained from the above reaction was filtered and rinsed repeatedly with deionized water and ethanol. Thereafter, the solid was transferred to a blast drying oven at 60°C and dried overnight to obtain the WS_2_/WO_3_ nanoparticles. Additionally, WO_3_ nanoparticles were obtained for comparison by adjusting the masses of C_2_H_2_O_4_·2H_2_O and thiourea to 0.63 and 0.8 g, respectively. The pH was adjusted to 2, while the other parameters remained unchanged (Figure 1).

### 2.3 Preparation of WO₃-CP and WS₂/WO₃-CP electrodes

The CP electrodes were alternately washed with deionized water and ethanol and then dried in a blast drying oven. 7.5 mg WO₃ and WS₂/WO₃ nanoparticles were added into a solution containing 60 μL 5 wt% Nafion solution, 340 μL deionized water, and 600 μL of absolute ethanol. The mixed solution was sonicated for 30 min to obtain the dispersions. The dispersions were systematically dropped onto the pre-treated CP electrodes. Finally, the modified CPs were dried to obtain the WO_3_-CP and WS₂/WO₃ - CP nanomaterials.

### 2.4 Selective enrichment and acclimation culture of microorganisms

To simulate the wastewater solution, the flow back fluid from shale gas pipelines was used in this present study. Five milliliter of flowback fluid was added to the Modified Postgate C (PGC) medium (Supplementary Table S1), followed by anaerobic cultivation at 30°C. After culturing in PGC media, the color of the enrichment medium turned dark black, confirming the presence of sulfate-reducing bacteria (SRB) and the production of a large amount of FeS (Widdel and Bak, 1992). Subsequently, the enrichment of SRB was further verified through microscopic observation and 16S testing. The enriched SRB mixed microbial community was then kept at a constant temperature incubator for acclimation, awaiting inoculation into the anolyte of the MFC.

### 2.5 Composition of anolyte and catholyte, and assembling of MFC

The crystal structures and high—resolution images of WO₃ and WS₂/WO₃ were analyzed by X-ray diffractometer (XRD), X-ray photoelectron spectroscopy (XPS), and high resolution transmission electron microscopy (HRTEM). The field emission scanning electron microscope (FESEM) equipped with an energy-dispersive X-ray (EDX) analysis device was used to observe the micromorphology of the modified electrodes. In addition, contact angle measurement (CA) was used to compare the hydrophilicity of different modified electrodes. During the test, a 10 μL deionized water droplet was dropped on the electrode surface, and the contact angle value of the droplet in a stable state was recorded.

Electrochemical impedance spectroscopy (EIS) tests were conducted using Gamry 1000 Electrochemical Analyzer (Warminster, PA, United States), with a sinusoidal voltage alternating amplitude of 10 mV, ranging from 10,000 to 0.01 Hz. Cyclic voltammetry (CV) tests were carried out using an electrochemical workstation CHI 660E (Shanghai Chenhua Instrument, China) in the range of −0.8~0.2 V vs. SCE at a scanning rate of 25 mV·s^−1^. All the electrochemical tests were performed in a traditional three electrode system, with the anode electrode in MFC as the working electrode, Pt sheet (1 × 1 cm) as the counter electrode, a saturated calomel electrode (SCE) as the reference electrode, and the anolyte solution was used as electrolyte. The concentration of SO4^2−^ was measured based on the absorbance of the solution using an ultraviolet spectrophotometer. One milliliter of diluted bacteria solution from different systems was added to 1 mL of conditioning reagent. To calculate the SO4^2−^ concentration using the standard curve, 60 mg of crushed barium chloride was used, vortex for 30 s, and the absorbance value was taken at 420 nm. In addition, the measurement of chemical oxygen demand in anode fluids was carried out using the dichromate method (GB 11914–89).

The removal rate of SO_4_^2−^ or COD can be expressed by Equation 1:


(1) $$\eta \%=\frac{\left(C_{0}-C_{1}\right)}{C_{0}}\times 100\%$$

Where the *η* represents the remote rate, *C*_0_ represents the initial concentration of SO_4_^2−^ or COD, and *C*_1_ represents the concentration of SO_4_^2−^ or COD at the point before refreshing the media.

The power of the MFC can be approximately obtained by adjusting the resistance via a resistance box as shown in Equation 2:


(2) $$P=UI=\frac{U^{2}}{R}\times 100\%$$

Where, U is the terminal voltage of the resistance and *R* is the resistance value of the resistance box.

## 3 Results and discussion

### 3.1 Characterization of WO_3_ and WS_2_/WO_3_ nanoparticle and their modified electrodes

**Figure 2:**
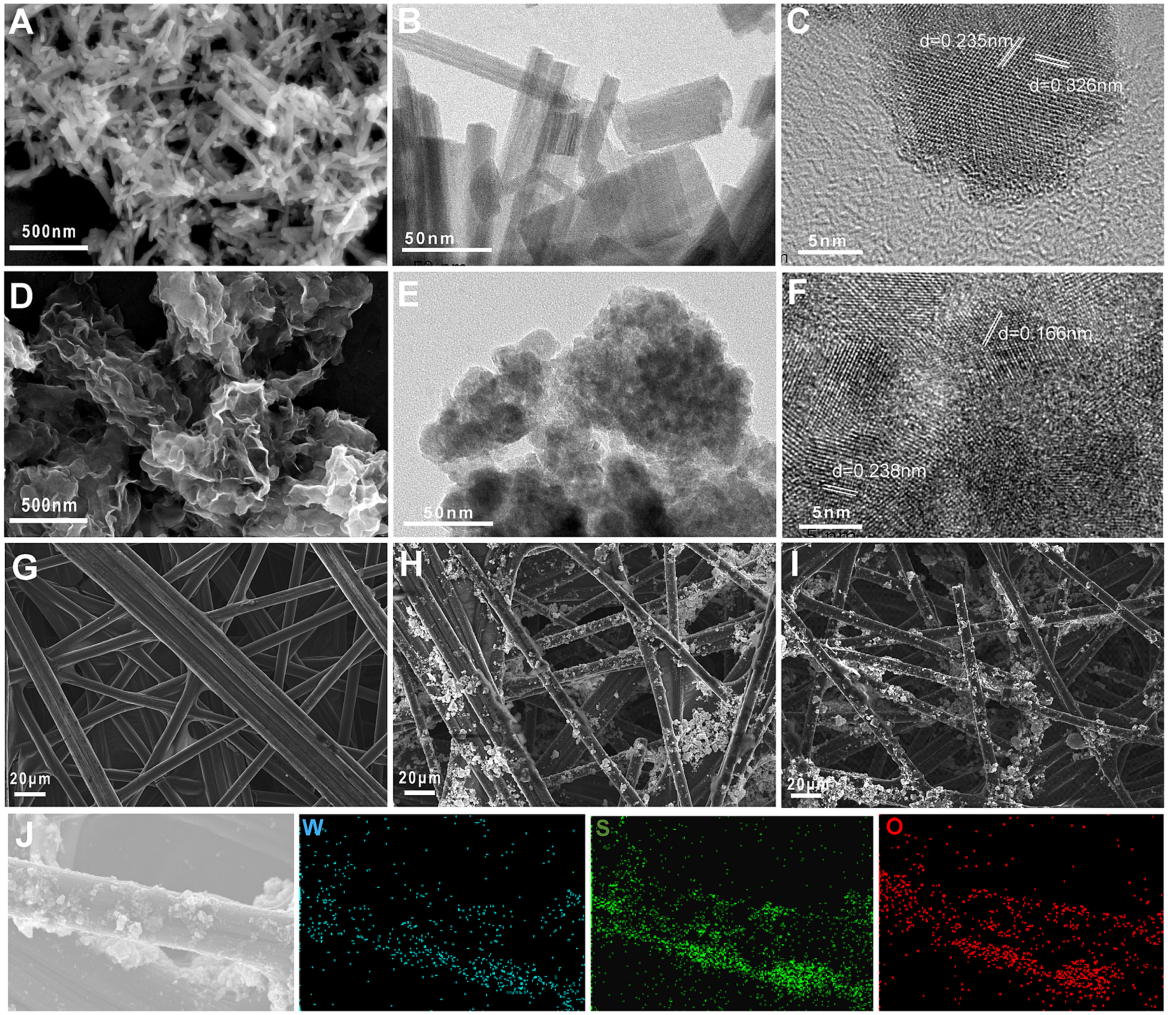
The micromorphologies of WO_3_ and WO_3_/WS_2_ nanoparticles: SEM images of WO_3_ **(A)** and WO_3_/WS_2_ nanoparticles **(D)**; the HRTEM images of WO_3_ **(B,C)** and WO_3_/WS_2_ **(E,F)**; the FESEM image of CP **(G)**, WO_3_-CP **(H)** and WO₃/WS₂-CP **(I)** and its elemental mapping image **(J)**.

The micromorphologies of WO_3_ and WS_2_/WO_3_ nanoparticle were shown in Figure 2. Figures 2A,B show that the prepared WO_3_ nanoparticles have a uniform rod-like structures, and the length of each nanorod is approximately 300 ~ 500 nm. In addition, the synthesized WS_2_/WO_3_ heterostructure microscopically presents a stacked nanosheet structure rather than a typical nanoflower or nanorod structure. This is because WO_3_ will affect the morphology of WS_2_ during the formation of its uniform three-dimensional structure (Figures 2D,E).

Phase identification and analysis of the HRTEM images of different materials were carried out using the Fast Fourier transform (FFT) method to obtain their crystal structure information. Figures 2C,F show the annotated HRTEM images of WO_3_ and WO_3_/WS_2_, respectively. For the WO_3_ nanoparticle, there are characteristic interplanar spacings of 0.235 nm and 0.326 nm, corresponding to the (210) and (200) crystal planes of the hexagonal crystal phase, respectively. In WS₂/WO₃, there are not only lattice fringes corresponding to the (103) crystal plane of hexagonal-phase WS₂ but also a characteristic interplanar spacing of d = 0.238 nm for WO₃ (210), which means that the WS₂/WO₃ heterostructure was successfully obtained. The difference in the interplanar spacing here compared with that of WO₃ might be due to the lattice distortion of WO₃ caused by the influence of the heterostructure. The crystal plane information mentioned above were further verified in the subsequent XRD analysis.

Figures 2G–I depicts the electrode morphology of bare CP electrode, WO_3_-CP, and WS_2_/WO_3_-CP, respectively. The unmodified bare CP consists of smooth carbon fibers interlaced with each other, while the two nano-modified materials are uniformly modified on the carbon fibers by the action of binder, which makes the surface rough and thus more conducive to microbial attachment. (Supplementary Table S2) summarizes the contents of the main elements in the two materials obtained from the corresponding EDX spectra. The composite material uniformly adhered to the surface of the CP, which was verified by elemental analysis in the mapping image (Figure 2J).

**Figure 3:**
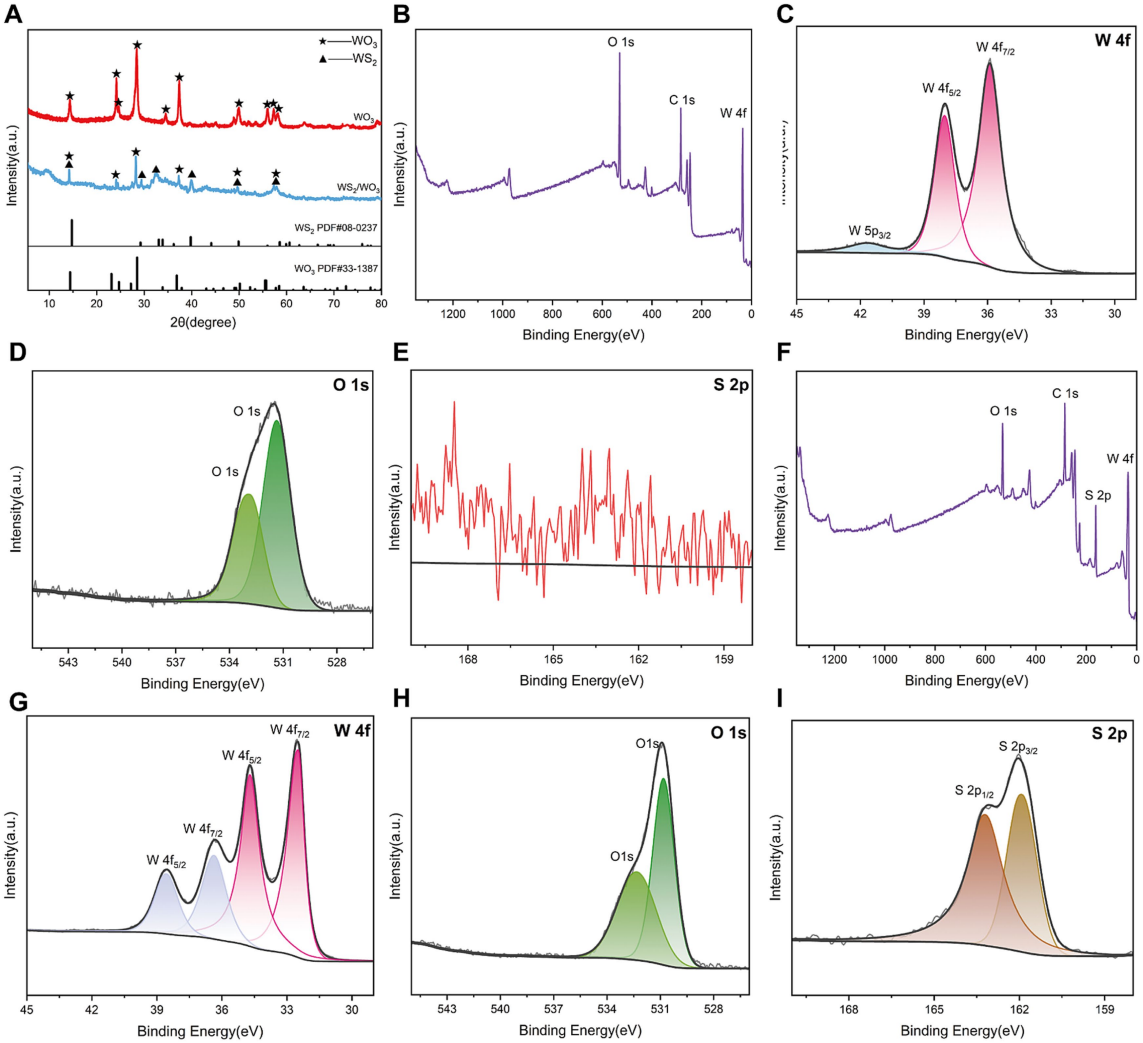
**(A)** XRD spectra of WO_3_ and WS_2_/WO_3_; **(B)** full XPS spectrum of WO_3_; **(C)** O 1 s and **(D)** W 4f and **(E)** S 2p spectrum of WO_3_; **(F)** full XPS spectrum of WS_2_/WO_3_; **(G)** O 1 s **(H)** W 4f and **(I)** S 2p spectrum of WS_2_/WO_3_.

The XRD images clearly show the phase and crystal structure information of the prepared powder (Figure 3A). The XRD pattern of pure WO_3_ matches with PDF#33–1,387, with distinct characteristic peaks observed at 14.11°, 24.45°, 28.19°, and 36.98°, corresponding to the (100), (110), (200), and (201) crystal planes of the monoclinic phase of WO_3_, respectively. For the synthesized composite material, in addition to corresponding to the known WO_3_ phase, the characteristic diffraction peaks located at 14.18°, 28.24°, and 39.58° in the XRD pattern also match with the (002), (004), and (103) peaks of the hexagonal phase of WS_2_ (PDF#08–0237). However, the weak intensity of the diffraction peaks in the pattern is attributed to the small particle size of the synthesized WS_2_/WO_3_ material.

XPS analysis was conducted to further understand the chemical composition and valence states of the elements in the prepared material. As expected, the XPS spectrum of WO_3_ did not detect any characteristic peaks for the S element, indicating that the material only involves chemical bonding between the W (Figure 3E) and O elements (Figure 3H). For WO_3_, the binding energies of 35.88 eV and 37.98 eV correspond to the W^6+^ position (Li et al., 2020). Figure 3G shows that WO_3_ well fits two typical characteristic peaks of O located at certain 530.83 and 532.33 eV positions, which are related to lattice oxygen in WO_3_.

Figure 3F displays the characteristic peaks of all elements present in WS_2_/WO_3_, primarily including peaks from C1s, O1s, W4f, and S2p, confirming the presence of O, W, and S elements in the sample. After deconvoluting the W4f peaks, two additional W4f peaks at 34.68 eV and 36.38 eV emerged for the composite material compared to pure WO_3_, which can be attributed to the 4f electron binding energy of W^4+^, suggesting that WS_2_ may also be present in the sample. Furthermore, the characteristic peaks appearing at 161.61 eV and 164.83 eV in the S2p spectrum further confirm the existence of WS_2_ in addition to WO_3_ in the material.

**Figure 4:**
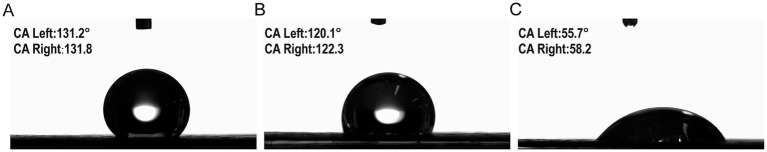
Contact angles of **(A)** CP, **(B)** WO_3_-CP and **(C)** WS_2_/WO_3_-CP.

Contact angle testing was used to evaluate the hydrophilicity of the material surface. The magnitude of the contact angle can reflect the adsorption ability of microorganisms on the electrode to a certain extent. For the unmodified substrate, the contact angle of about 131.2° (Figure 4A) showed weak hydrophobic properties, which is unfavorable for the adhesion of anode microorganisms in the MFC. In addition, it was observed that the contact angle of the CP surface modified by WO_3_ stabilized at 121° within a short time. Compared with CP, the hydrophilicity of modified WO_3_-CP was slightly enhanced. It is worth noting that when the WS_2_/WO_3_-CP electrode just came into contact with the droplet, the contact angle showed a significant decrease, and its instantaneous contact angle reached 55.7°, indicating that the hydrophilicity of the composite nanomaterial was further improved. This sharp decrease in the contact angle may be due to the fact that the large specific surface area of nanoscale WS₂ itself brings a large number of active sites, and the uneven surface charge distribution attracts polar water molecules. Figures 4A–C indicated that the WS_2_/WO_3_-CP has a rougher surface structure than the original CP electrode and the WO_3_-CP electrode. As a high-performance anode of the MFC, those modified electrode with rougher surface were considered to have high biocompatibility than the origin ones.

### 3.2 Electrochemical tests

In order to explore the electrochemical performance of different electrodes, the electrochemical properties were characterized using CV and EIS tests. CV is a common method for evaluating the electrochemical activity and catalytic performance of electrodes The properties of modified electrode can be reflected from current density, redox peaks via CV curves.

**Figure 5:**
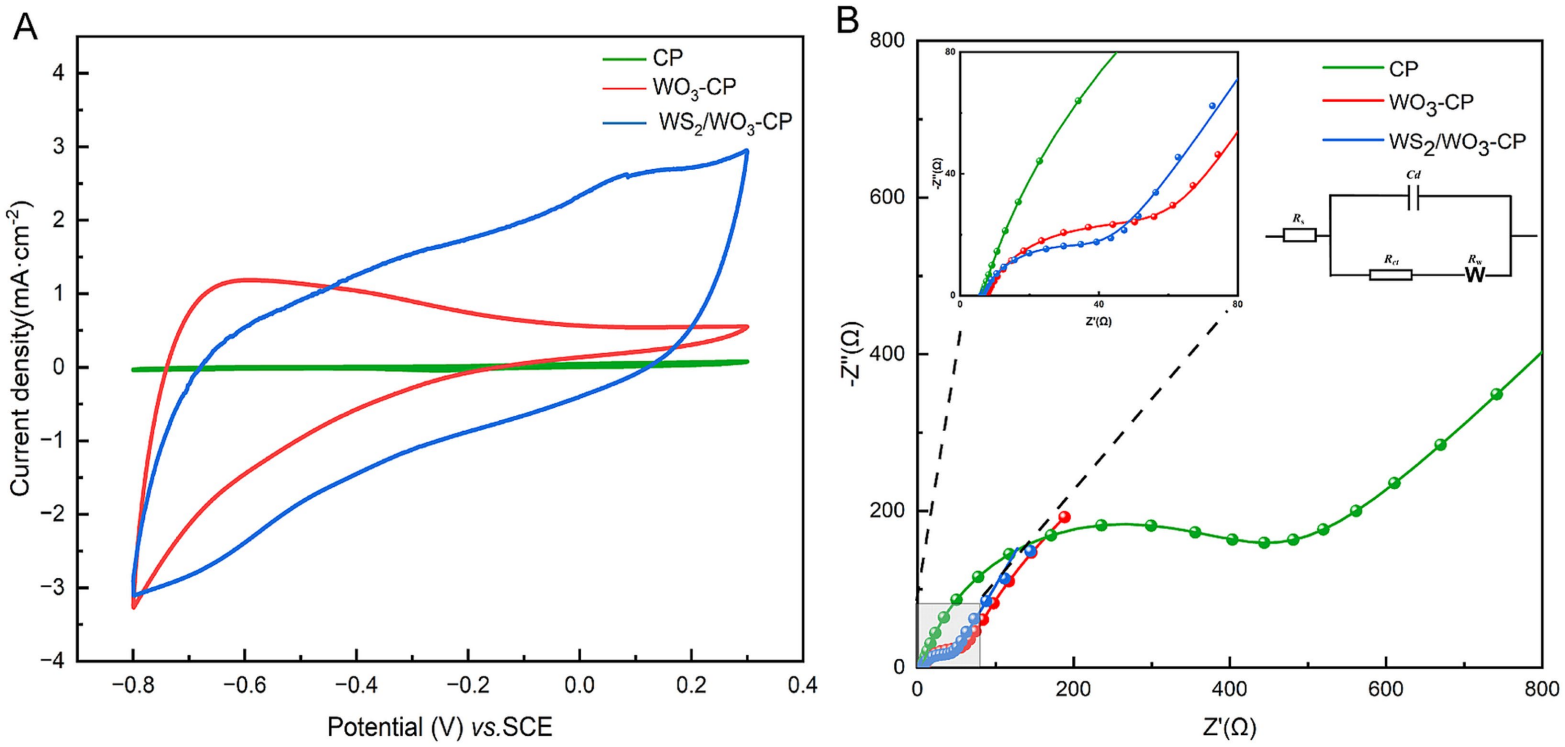
**(A)** CV curves and **(B)** EIS curves of different electrodes.

In this CV test, fresh anolyte was selected as the electrolyte, and three electrodes with the same area were tested at a scanning rate of 25 mV/s. The results showed (Figure 5A), compared with the WO_3_-CP and CP electrodes, the WS_2_/WO_3_-CP electrode exhibits a larger current density and area and a higher number of electrons involved in redox. This indicates that the WS_2_/WO_3_-CP electrode has a higher electrochemical active area, so it can have more superior electrical conductivity. This is crucial for the attachment of EAMs to the anode surface and facilitating the EET. In addition, in terms of shape, none of the three electrodes showed obvious redox peaks, indicating that the electrodes themselves hardly undergo redox reactions in this defined anolyte solution.

In addition, the EIS results of the three types of anodic electrodes in a mixed solution containing 5 mM potassium ferricyanide and 5 mM PBS were compared, the charge transfer resistance (*R*_ct_) were obtained based via fitted Nyquist curves (Figure 5B). As can be seen from Table 1, the *R*_s_ among the three electrodes are roughly the same, but the unmodified CP electrode has the largest *R*_ct_, which is approximately 425.2 Ω. The WO_3_-CP and WS_2_/WO_3_-CP electrodes have *R*_ct_ values of 45.18 and 37.51 Ω, respectively, which are significantly lower than that of the CP electrode. Therefore, it can be considered that the addition of WS_2_ reduces the resistance of the material to a certain extent, enabling the WS_2_/WO_3_-CP electrode to have a higher electron transfer efficiency, and thus facilitated the electron transfer of microorganisms on the electrode surface less hindered.

**Table 1:** Fitting results for different anodes based on Nyquist plots.

Electrodes	*R*_s_(Ω)	*Cd*×10^−4^ (Ω^−1^ s^n^)	*n*_f_	*R*_ct_(Ω)	*W* (Ω^−1^)
CP	6.33	1.36	0.8380	425.40	0.00421
WO_3_-CP	7.79	1.54	0.8805	45.18	0.01816
WS_2_/WO_3_-CP	6.43	17.44	0.8854	37.51	0.02407

**Figure 6:**
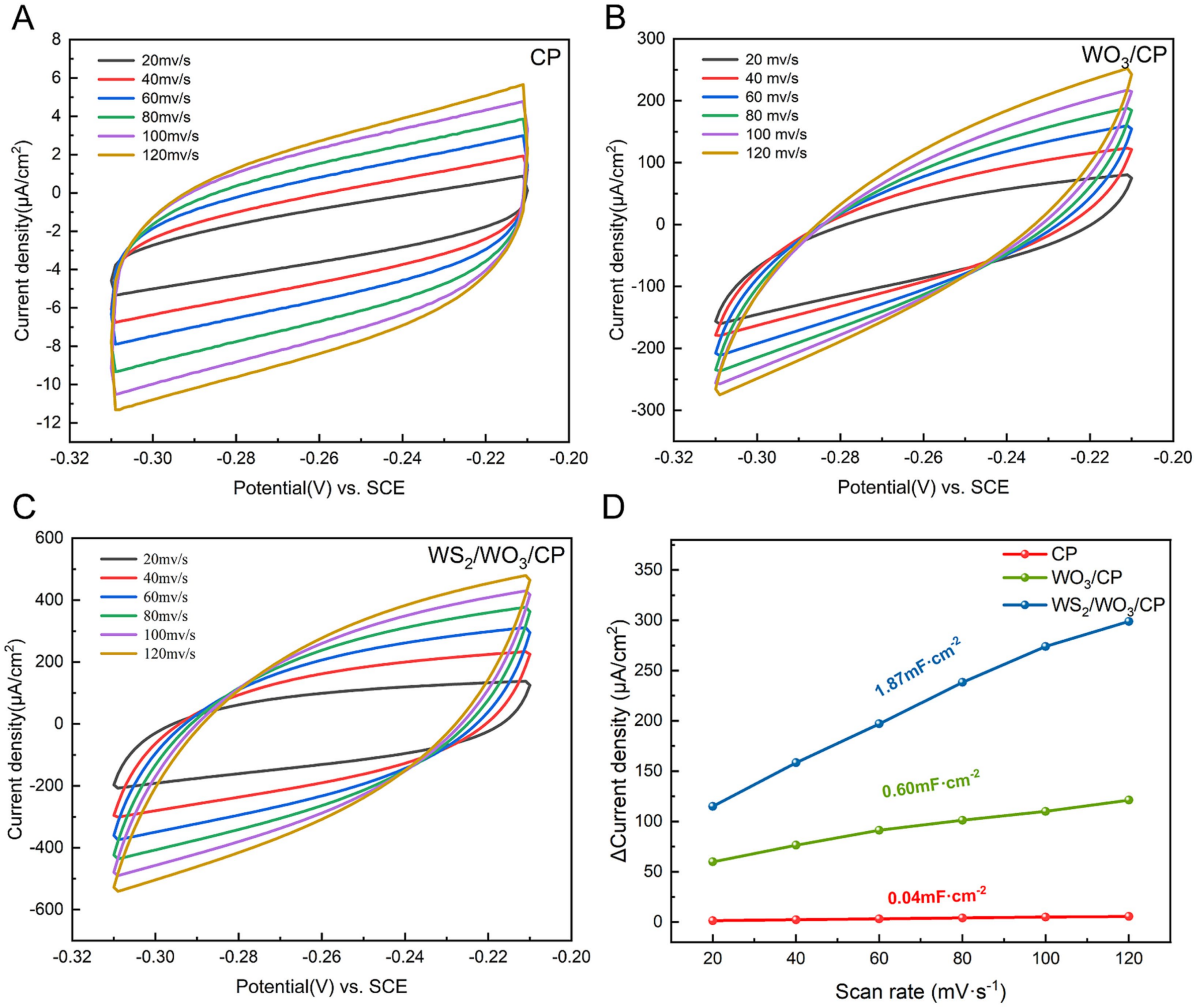
CV curves for **(A)** CP, **(B)** WO_3_-CP, and **(C)** WS_2_/WO_3_-CP in anode electrolytes with various scan rates; **(D)** Linear relationship between bilayer current density and scanning rate for different electrodes.

The cyclic voltammetry curves of CP, WO_3_/CP and WS₂/WO_3_-CP at different scan rates from 20 mV/s ~ 120 mV/s are shown in Figures 6A–C, respectively. In general, the shape and current response of the cyclic voltammetry curves reflect the electrochemical activity and reaction kinetics of the materials. The curve area of CP is relatively small and the increase of the current density with the scan rate is limited. In contrast, the curves of WO₃-CP and WS₂/WO₃-CP show larger electrochemical areas and higher current densities, which are attributed to the enhanced electrochemical activity of the modified electrode with WO₃ and WS₂/WO_3_. In addition, the magnitude of the electrochemically active area can be estimated by comparison of electrochemical double layer capacitance (*C*_dl_). Figure 6D presents the variation of current density versus scanning speed, and the slope of the fitted straight line responds to the magnitude of *C*_dl_ of the electrode in the test solution. According to the calculation, the largest magnitude of *C*_dl_ is found in WS₂/WO_3_-CP (1.87 mF·cm^−2^), followed by that of WO_3_-CP electrode (0.60 mF·cm^−2^). While the smallest *C*_dl_ is found in CP (0.04 mF·cm^−2^). The results suggests that surface modification of the electrodes by WS_2_/WO_3_ effectively improves the charge storage capacity and electroactive area of CP electrode.

### 3.3 Performance analysis of MFC

**Figure 7:**
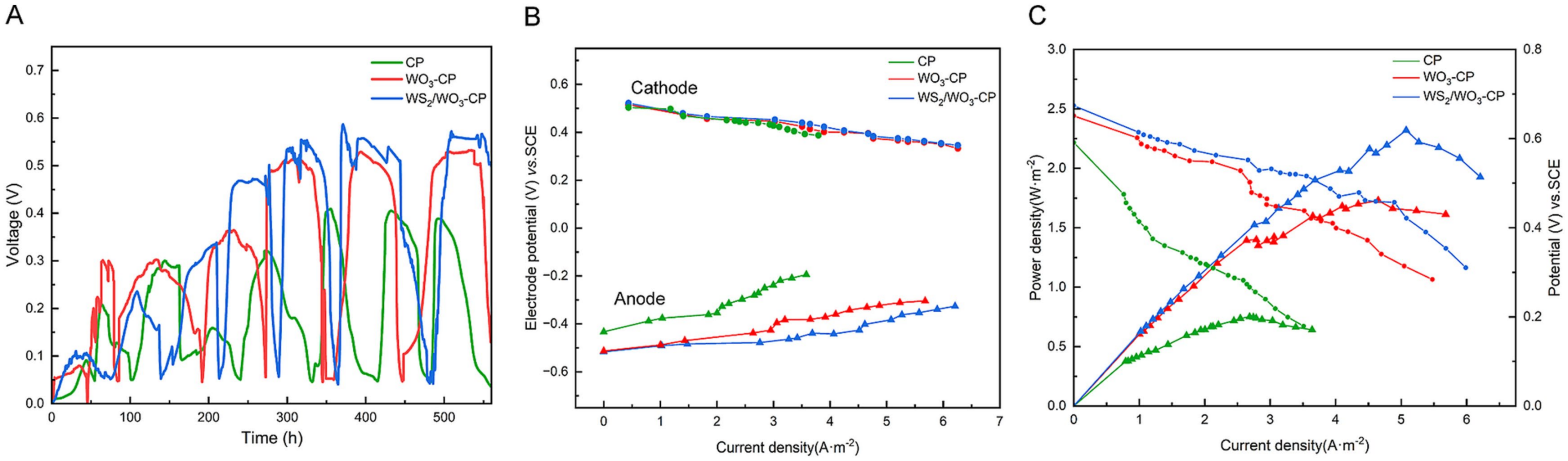
**(A)** Voltage output, **(B)** the trend of electric potential with current density, and **(C)** the power density and the polarization curves of MFCs.

Three systems of dual-chamber MFCs were constructed with carbon brushes as cathodes, CP, WO_3_-CP, and WS_2_/WO_3_-CP as anodes, respectively. The output performance of the MFC voltage was recorded in real time. The anolyte was replaced if the voltage dropped to approximately 0.05 V, and the voltage recording was continued until the highest output voltage remained stable enough over multiple cycles. As can be seen from Figure 7A, during the stable stage of MFC output voltage, the MFC with WS_2_/WO_3_-CP as the anode had an output voltage of approximately 0.556 V, which was higher than that in MFCs with WO_3_-CP (0.528 V) and CP (0.394 V) as anodes. This result corresponded to the previous results. This is because the rough and hydrophilic surface gives WS_2_/WO_3_-CP the ability to attract microorganisms to attach to its surface, as well as excellent electrochemical properties to facilitate the EET process on the electrode surface.

During the period when the MFC stably operates and achieves its highest voltage output, the external resistance is systematically adjusted via a resistance box. Simultaneously, the electrode potential and the terminal voltage of the resistance box under various resistance values are meticulously recorded. Thus, the current density value can be got at different under various resistance values. Then, the power density and polarization curves were plotted based on the Equation 2. Figure 7B shows the trend of electric potential with current density. As to the cathodic electrode, the potential in the three systems groups of MFCs showed a similar downward trend, and the variation range was small, indicating that the output differences of the MFCs were not caused by cathode polarization. However, the potential changes of different anodes were clearly distinguishable in Figure 7B. In particular, the CP electrode produced the highest potential increase within the variation range of a small current density. For the WS_2_/WO_3_-CP electrode and the WO_3_-CP electrode, the potential changes occurred in a gentler manner, indicating that the two have higher electrochemical stability.

The polarization and polarization curves of the three MFCs are shown in Figure 7C. The MFC equipped with the WS₂/WO₃-CP electrode showed the highest power density output, approximately 2.32 W·m^−2^, which is higher than the maximum power densities of the MFCs with WO₃-CP (1.73 W·m^−2^) or CP (0.75 W·m^−2^) as the anode. The power density of WS_2_/WO_3_-CP is about 3.09 times that of CP, indicating that the modified has more excellent power generation performance than CP. As can be seen from the electrode polarization curves, the MFCs with WS_2_/WO_3_-CP and WO_3_-CP as anodes show minor voltage changes under a relatively high current density (approximately 6 A·m^−2^). This indicates that, compared with the MFC using a CP electrode as the anode, these two MFCs with modified electrodes have higher anti-polarization performance and internal resistance.

**Figure 8:**
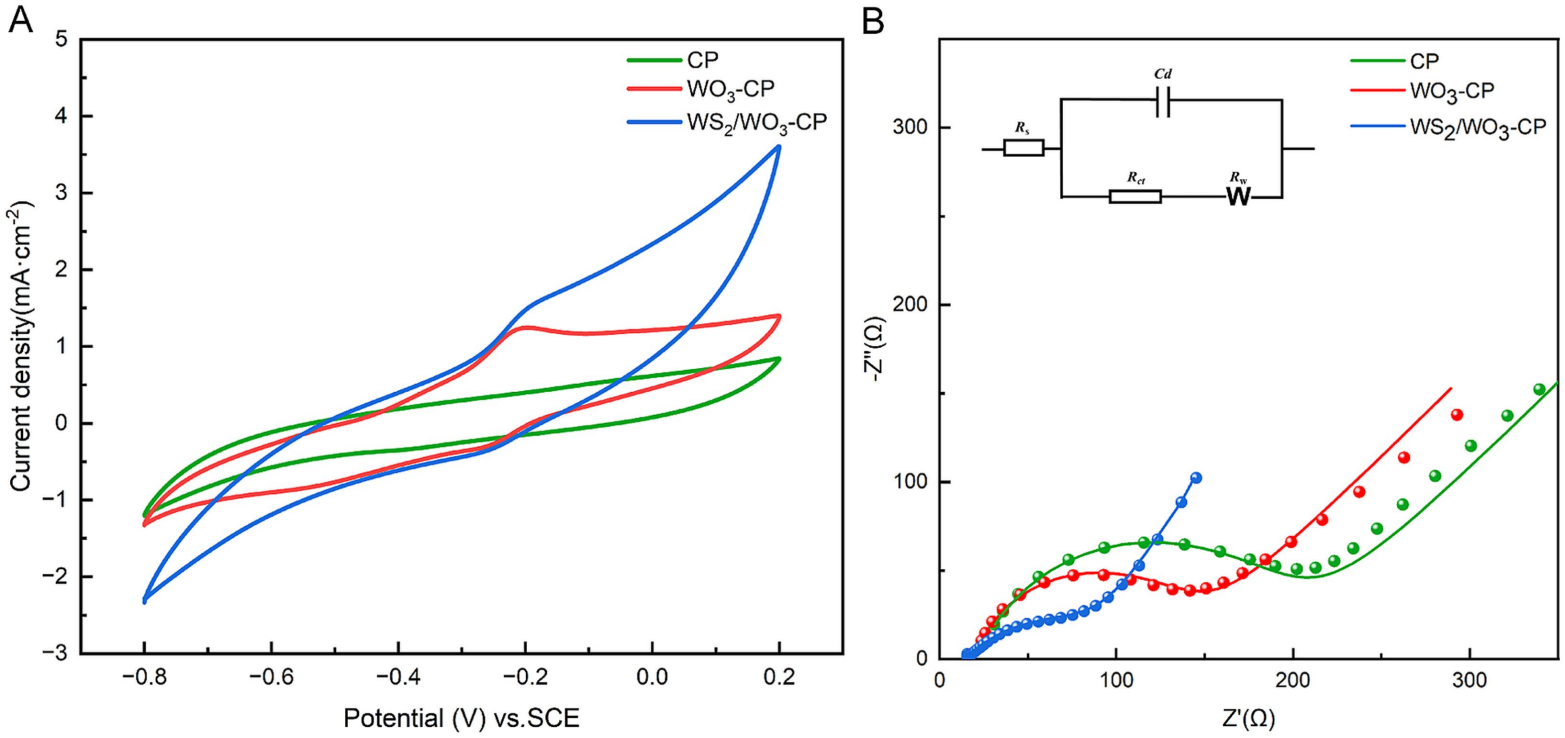
**(A)** CV curves of different bioanodes; **(B)** EIS curves of different bioanodes.

Electrochemical analysis of the bioanode helps to further clarify the differences in output performance among different MFCs. Here, both CV and EIS tests were conducted in the anolyte when the voltage output was stable, and other conditions were the same as those in the previous tests. As depicted in Figure 8A, when transitioning from the CP bioanode to the WO_3_-CP bioanode and then to the WS_2_/WO_3_-CP bioanode, there is a progressive enlargement of the curve areas in the CV tests, indicating that the electrical conductivity and capacitive characteristics are successively enhanced. Meanwhile, obvious redox peaks appear at-0.251 V and-0.210 V on the CV curves of the WO_3_-CP bioanode and the WS_2_/WO_3_-CP bioanode. The appearance of redox peaks may be caused by the EET process caused by attached microorganisms on electrode surface. During this process, the electron carriers or outer-membrane cytochrome C in the microorganisms undergo a conversion between the oxidized and reduced states (Jain et al., 2011, 2012; Yang et al., 2024). Thanks to the excellent biocompatibility of the modified materials, a large number of EABs are enriched on the electrode surface, which in turn improves the efficiency of EET between microorganisms and electrons.

The fitted results shown in Figure 8B and Table 2 indicate that the largest *R*_ct_ (179.30 Ω) is found in CP bio-electrode, followed by that in WO_3_-CP (123.00 Ω) and WS_2_/WO_3_-CP (75.58 Ω) bio-anodes, respectively. Therefore, there is no doubt to find that during the operation of the MFC, the WS₂/WO₃-CP bio-electrode exhibits the highest charge transfer efficiency and superior catalytic performance.

**Table 2:** Fitting results for different bioanodes based on Nyquist plots.

Electrodes	*R*_s_ (Ω)	*C*_d_ × 10^−4^ (Ω^−1^ s^n^)	*n*_f_	*R*_ct_ (Ω)	*W* (Ω ^−1^)
CP	21.53	2.98	0.7739	179.30	0.01608
WO_3_-CP	19.33	4.62	0.8074	123.00	0.01845
WS_2_/WO_3_-CP	10.41	22.42	0.5299	75.58	0.01677

Excessive COD and sulfate in wastewater can cause damage to the water environment (Lee et al., 2014). However, anode microorganisms dominated by SRB in MFCs have been proven to effectively degrade these two pollutants simultaneously and generate electricity (Miran et al., 2021). In light of this, we conducted an assessment of the COD and sulfate removal capacities of three distinct types of MFCs.

**Figure 9:**
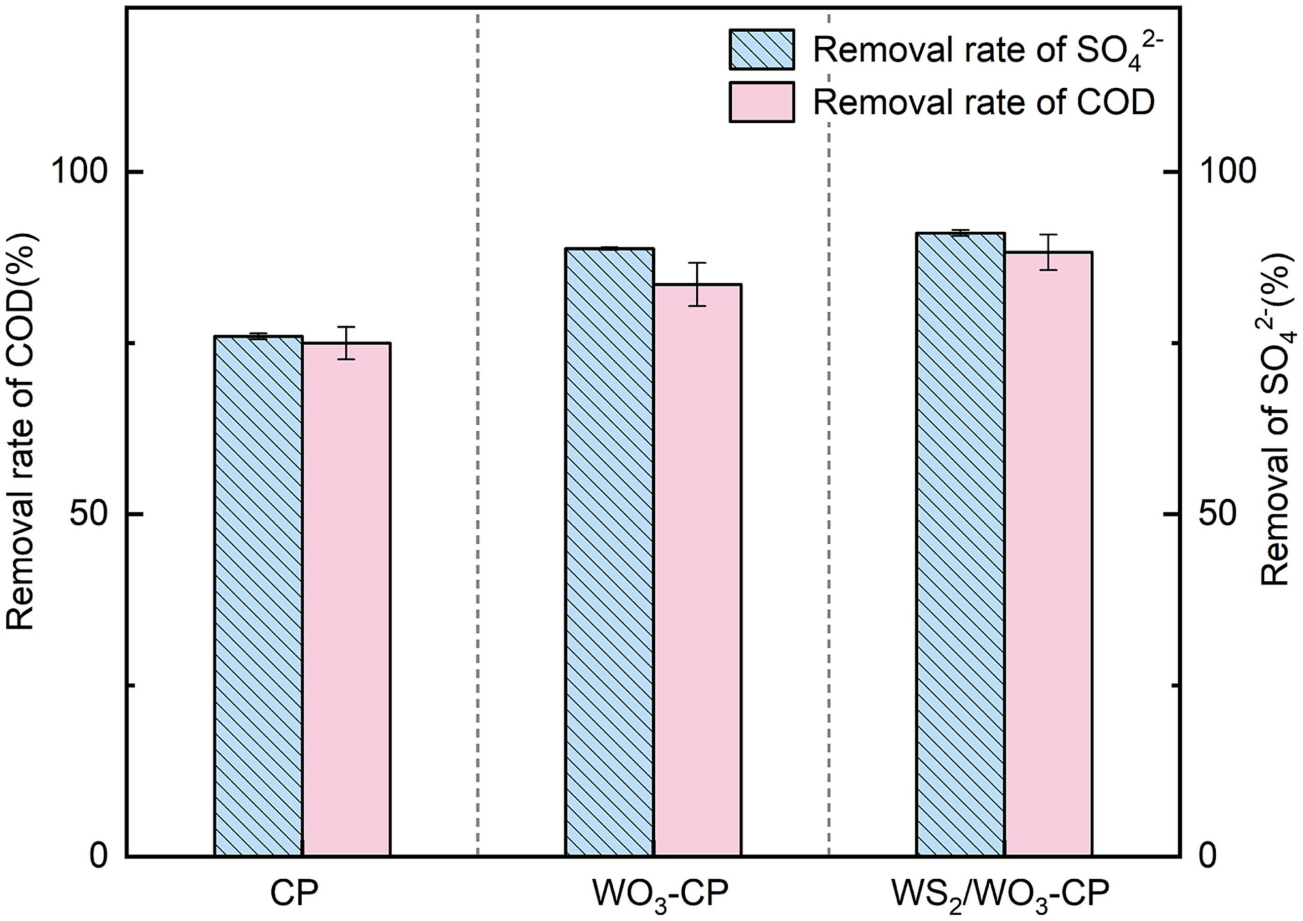
The removal rate of COD and sulfate in MFCs equipped with different anodes.

As can be seen from Figure 9, the removal rates of SO₄^2−^ in the MFCs equipped with WO_3_-CP and WS_2_/WO_3_-CP electrodes reached 88.79 and 91.07%, respectively. In contrast, the SO₄^2−^ removal rate of MFC with CP electrode was 76.00%. In terms of COD removal, the three groups of MFCs showed a similar trend to that of SO₄^2−^ degradation, but the overall removal rates were slightly lower than those of SO_4_^2−^. Among them, the COD removal rate of the MFC with the WO_3_-CP anode reached 83.57%, while the MFC with WS_2_/WO_3_-CP anode achieved a COD removal rate of 88.27%, which was 17.69% higher than that of the MFC with the CP electrode.

### 3.4 Morphology of attached biofilm on the anode surface and microbial community

**Figure 10:**
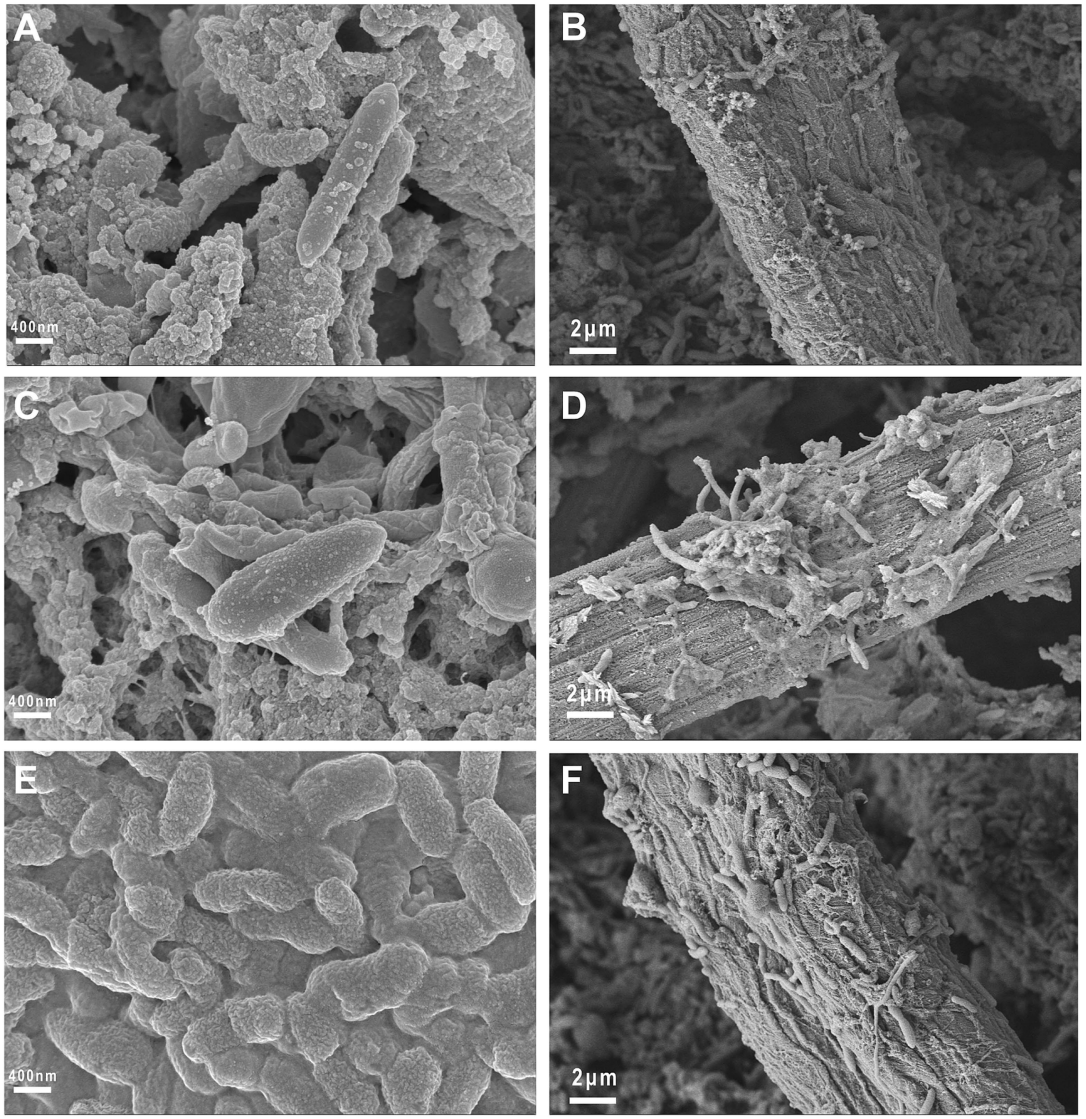
Morphology of the biofilm on the anode surface. **(A,B)** FESEM images of the biofilm on the CP electrode; **(C,D)** FESEM images of the biofilm on the WO_3_-CP electrode; **(E,F)** FESEM images of the biofilm on the WS_2_/WO_3_-CP electrode.

After the electrochemical tests were completed, FESEM was used to observe the morphology of the attached biofilm on the electrode surface. The involvement of EAM-embedded biofilms in extracellular electron transfer with electrodes can be achieved via either direct physical contact or redox-mediated indirect pathways. As shown in Figure 10, rod-shaped and elongated microorganisms were present on the surface of the carbon fiber, with a length of approximately 1.5 ~ 3 μm. It can be seen that a denser biofilm adhered to the WS_2_/WO_3_-CP electrode compared to the WO_3_-CP and CP electrodes, indicating that this electrode has better biocompatibility.

### 3.5 Analysis of the mechanism of electricity production

**Figure 11:**
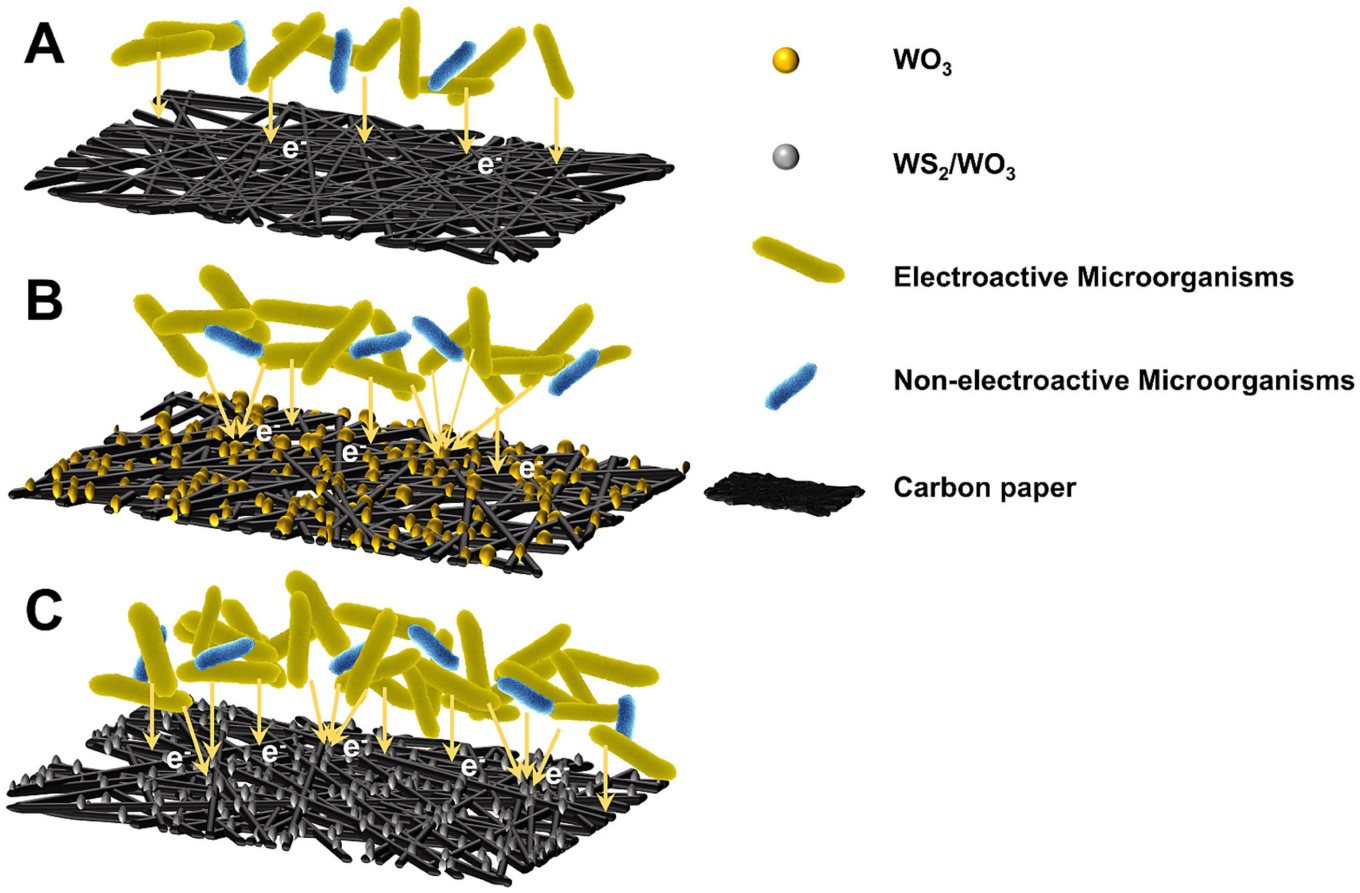
Electron transfer mechanisms between electroactive microorganisms and electrodes: **(A)** CP; **(B)** WO_3_-CP; **(C)** WS_2_/WO_3_-CP.

The observed discrepancies in power output among MFCs employing distinct electrode configurations can be mechanistically interpreted through the proposed electron transfer framework (Figure 11). Primarily, the homogeneous deposition of catalytic layers on carbon paper substrates significantly enhances both surface roughness and hydrophilic characteristics. The nanomaterials on the electrode surface can increase the specific surface area of the original electrode and the number of redox reactive sites, while their inherent biocompatibility favors more microorganisms, especially EAMs, to form biofilms and carry out electron transfer on the electrode surface. Notably, Some recent studies (Wang et al., 2018; Kim et al., 2023) have found that the heterostructured materials excel in synergistically enhancing the electron transfer efficiency and reducing the internal resistance of charge transfer, and the EAMs adhering to such materials accelerate the oxidative degradation of organic substrates, resulting in a significant enhancement of the catalytic activity on the electrode surface. In summary, since the WS₂/WO₃-CP electrode attracts the largest number of microorganisms, the dense biofilm ensures that the EAMs are not easy to be dislodged from the electrode surface. In addition, the excellent electrocatalytic activity of WS₂/WO₃ will synergize with the EAMs to rapidly transfer the electrons from organic pollutants to the external circuit, which results in more excellent power density and output performance of MFCs assembled with WS₂/WO₃-CP as anode.

## 4 Conclusion

In this study, WS_2_/WO_3_ and WO_3_ composite materials were prepared via a simple hydrothermal synthesis method. Subsequently, these composites were integrated with carbon paper (CP) to develop WS_2_/WO_3_-CP and WO_3_-CP anodes. These anodes demonstrated remarkable performance in removing COD and sulfate from wastewater, achieving removal rates of 88.27 and 91.07% respectively, which were 13.27 and 15.07% higher than those attained by unmodified CP anode. Subsequently, the WS_2_/WO_3_-CP anode exhibits good hydrophilicity and high electrical conductivity. The synergistic effects can also enhance the substrate’s ability to attract microbial attachment and promote extracellular electron transfer near the anode. The maximum power density of the MFC equipped with the WS_2_/WO_3_-CP anode reached 2.32 W·m^−2^, which is higher than that of the MFCs equipped with WO_3_-CP (1.73 W·m^−2^) and CP (0.75 W·m^−2^) anodes, and was also superior to that of some carbon-based composites (Zhong et al., 2018; Lou et al., 2021). In summary, the WS₂/WO₃ nanomaterials prepared in this study show promising propertied for power generation and pollutant degradation in MFCs. The low cost and process simplicity of the hydrothermal synthesis method gives it potential for practical applications. The performance and economy can be further improved in the future by optimizing the material ratios or performing theoretical calculations on electrochemical reactions.

## Data Availability

The raw data supporting the conclusions of this article will be made available by the authors, without undue reservation.
