# Supplementary material for: WS2/WO3 modified carbon anode as efficient electrocatalysts for enhancing electricity generation and pollution removal
Source: Front Microbiol. 2025 Apr 28;16:1589441. doi: 10.3389/fmicb.2025.1589441 (PMC12066612; doi:10.3389/fmicb.2025.1589441)
Supplement: Supplementary file 1 [file Table_1.docx]

**Electronic Supplementary Material**

**WS_2_/WO_3_ modified carbon anode as efficient electrocatalysts for enhancing electricity generation and pollution removal**

Yugang Sang ^1,2^, Quantong Jiang ^1,2,3*^, Fang Guan ^2,3*^, Nan Wang ^2,3^, Ini-Ibehe Nabuk Etim ^2,3^, Keliang Fan ^2,4^, Jizhou Duan ^2,3^

^1^ Department of Materials Science and Engineering, Qilu University of Technology, Jinan, 250353, China

^2^ State Key Laboratory of Advanced Marine Materials, Institute of Oceanology, Chinese Academy of Sciences, Qingdao, 266071, China

^3^ Guangxi Key Laboratory of Marine Environmental Science, Institute of Marine Corrosion Protection, Guangxi Academy of Sciences, 98 Daling Road, Nanning 530007, China

^4^ Department of Bioengineering, Qilu University of Technology, Jinan, 250353, China

*** Correspondence:**Fang Guan and Quantong Jiang
[guanfang@qdio.ac.cn](mailto:guanfang@qdio.ac.cn) and jiangquantong@qdio.ac.cn

**Table S1.** Media formulation for enrichment of sulfuric acid-reducing bacteria

| Component | Concentration ( /L) |
| --- | --- |
| KH_2_PO_4_ | 0.5g |
| NH_4_Cl | 1g |
| CaCl_2_ | 0.1g |
| MgSO_4_·7H_2_O | 2g |
| NH_4_Fe(SO_4_^2-^)_2_ | 1g |
| Yeast extract | 1g |
| 70% Sodium lactate solution | 7ml |

**Table S2.** The contents and atomic ratios of the main elements in the two materials obtained from the corresponding EDX spectra

|  | Contents in WO_3_-CP ( wt.%) | Contents in WS_2_/WO_3_-CP ( wt.%) |
| --- | --- | --- |
| C | 73.727 | 50.4 |
| O | 7.58 | 5.203 |
| S | 0.315 | 5.958 |
| W | 18.379 | 38.439 |
